# Assessment of the Number of Phlebotomists in a Newly Established Sample Collection Center

**DOI:** 10.7759/cureus.43323

**Published:** 2023-08-11

**Authors:** Sindhu Toomukuntla, Chandra Vamshi Vemula, Parag Patil, Shrinivas B Somalwar, Gunvanti Rathod, Shailaja Prabhala

**Affiliations:** 1 Pathology and Lab Medicine, All India Institute of Medical Sciences, Bibinagar, Bibinagar, IND

**Keywords:** phlebotomy waiting time, sample collection centre, estimated capacity of phlebotomy centre, blood draw time, phlebotomist

## Abstract

Introduction: Phlebotomy, i.e., the collection of blood samples, is one of the most commonly performed procedures in almost all hospital settings. The phlebotomy center is the first point of contact for patient samples with the laboratory services. The patient load visiting the phlebotomy center of a rapidly developing hospital is very variable and unpredictable. This leads to staffing issues related to a number of phlebotomists. The actual phlebotomy procedure requires only a few minutes, but the total time includes the patient's arrival to departure from the phlebotomy center. In this study, we have attempted to assess the adequacy of the number of phlebotomists in our sample collection center and to determine how many patients can be attended to comfortably by each phlebotomist. As the sample load increases, the burden on phlebotomists also increases, and they may or may not express the strain of it. We attempted to determine the cut-off patient numbers above which request for additional personnel has to be put into the hospital administration.

Materials and methods: This was a prospective, hospital-based, observational study carried out in the outpatient sample collection center section at the All India Institute of Medical Sciences, Bibinagar, Telangana, over a period of one month, i.e., December 2022. The movement of 1200 patients was observed for the phlebotomy procedure. Patient details, the time taken for registration, waiting time, and phlebotomy time were noted, along with the hindering factors in the phlebotomy center.

Observations and results: There were 680 males and 520 females. The mean time for patient arrival to departure from the phlebotomy center and the mean waiting time was 9.8 minutes and 6.5 minutes, respectively. Various reasons for increased phlebotomy time were pediatric patients, anxious patients, postprandial sample patients, difficulty in finding veins, etc. Though the estimated capacity of the phlebotomy center is apparently satisfactory with four personnel, many hidden causes for time loss were observed.

Conclusion: An adequate number of trained and effective phlebotomists is the first step in ensuring the success of any laboratory service, and while deciding on this "adequate number," not only the direct effort, but also the indirect effort, operational needs and emergencies have to be kept in mind. Each phlebotomist in a six-hour shift can comfortably attend 30 to 35 outpatients for phlebotomy. When this number exceeds it, additional staff has to be added. Adopting measures to reduce the waiting time for phlebotomy procedures will improve the phlebotomy center's service. The study provides a basis for the modification of a number of phlebotomists in order to ensure optimal patient service.

## Introduction

Phlebotomy is one of the most frequently done laboratory procedures in health care [1]. The patient load in a phlebotomy center varies on an hour-to-hour and day-to-day basis. This gives rise to staffing issues, and it is difficult to decide on the exact number of personnel to be placed at this site. For the health care providers and the patients, it is desirable to have a minimum waiting time in this area. Focusing only on the phlebotomy procedure to decide upon the number of phlebotomists will lead to understaffing and affect downstream sample testing and results.

There are various masked factors, like infants, old patients, wrongly filled requisition forms, etc., inbuilt in the phlebotomy work area that increase the time for the same. An improperly collected blood sample will give incorrect results and necessitate repeat sample testing. Almost 70% of errors occur in the pre-analytical phase [1,2]. The pre-analytical process is defined as “steps starting in chronological order from the clinician’s request, including the test requisition, preparation of the patient, collection of the primary sample, transportation to and within the laboratory and ending when the analytical examination starts.” One of the factors contributing to this issue is inadequate laboratory personnel, especially in resource-poor settings. Developed countries have a lower disease burden and more health workers. However, a less-developed area like Africa has about 24% of the global burden of disease but only 3% of the global health personnel [3]. This irregular distribution of qualified health workers is a longstanding issue, especially in the rural areas of various countries [3,4].

Quality in laboratory services is the guarantee that every single step throughout the total testing process has been performed correctly and satisfies the needs and expectations of the customer/patient [5,6]. The quality of medical laboratories mainly refers to technical or analytical quality and is assessed by an indicator termed turnaround time (TAT) [7]. TAT consists of pre-analytical, analytical, and post-analytical phases. For laboratory personnel, it would be from the time of receipt of the sample until the generation of the report, whereas for the clinician, it is the time taken from the raising of the test requisition until the report reaches him/her. Decreasing the waiting time for phlebotomy could shorten the TAT. Often, phlebotomy takes less time than waiting [8]. The present study was focused on determining if the number of phlebotomy personnel is adequate for the present patient load in a newly established rural care setting and also determining the cut-off above which new personnel should be added. It also aimed to find the number of phlebotomy samples that could be collected per working shift by a laboratory technician. It is an attempt to help identify the possible barriers to efficient phlebotomy services and also aids in making recommendations to overcome them.

## Materials and methods

This was a prospective, hospital-based, observational study carried out in the central sample collection center section under the Department of Pathology and Lab Medicine at the All India Institute of Medical Sciences, Bibinagar, Telangana, over a period of one month, during December 2022. The study was approved by the Institute Research Committee and the Institute Ethics Committee, AIIMS, Bibinagar, with approval/clearance letter number: IEC Ref No.: AIIMS/BBN/IEC/OCT/2022/228.

A total of 1200 outpatient department (OPD) patients coming to the sample collection center were observed for this study. Their movement, from the time the patient reached the center to the time the patient exited the center, was observed. Observations were made on weekdays during the morning hours at the sample collection center. Patients' movements were observed irrespective of their gender, age, or differently abled status. Physically challenged or wheelchair-bound patients were also observed in this study, as their movement also influenced the time spent in the center. Patients with incomplete request forms, indoor patients, and patients coming for COVID-19 testing were excluded from this study.

In the initial step, the time taken for registration was noted, which was considered step 1. This included the interaction of the patients with the nursing officer who documented the patient details manually in the sample collection register. Then step 2 time was noted, wherein the patients waited in a queue for their turn to reach the phlebotomy station, which was immediately adjacent to the registration counter. Then step 3 time was noted, which was the time duration of the actual phlebotomy procedure, starting from when the patient sits in the phlebotomy chair until he/she leaves the phlebotomy chair.

The investigators observed and noted down the time taken for each patient's phlebotomy procedure under the heading of the above three steps. The time was noted in seconds with the help of a stopwatch and later converted to minutes. The study did not permit any interaction of the investigators with technicians, their assistants, nursing officer, or patients. It was purely an observational study.

The collected data were entered in a Microsoft Excel sheet (Microsoft Corporation, New York, USA), and the results are expressed in terms of frequency and percentage.

## Results

The sample collection center, to begin with, had two technicians who handled patient registration, tube labeling, and actual phlebotomy procedures. Gradually, as the number of patients increased and crossed 90 to 100 patients per day, each technician performed about 50 phlebotomies per six-hour shift (eight to ten minutes per procedure, including the registration and labeling effort), and three nursing assistants were added to phlebotomy center. The center had four sample drawing stations, each manned by one phlebotomist/nursing assistant and the third nursing assistant manned the registration counter. The assistant checked the request forms, made the necessary entries in the registers, and then the patient moved towards the phlebotomy table, where the phlebotomist drew the sample(s).

A total of 1200 patients were observed who came to the outpatient sample collection center for phlebotomy procedures. There were 680 male and 520 female patients, and the male-to-female ratio was 1.3:1 (Table 1).

**Table 1:** Age and gender-wise distribution of patients.

Age in years	Males	Females	Total
0-10	51	39	90 (7.5%)
11-20	116	97	213 (17.7%)
21-30	85	88	173 (14.4%)
31-40	117	84	201 (16.7%)
41-50	120	92	212 (17.6%)
51-60	113	74	187 (15.5%)
61-70	50	31	81 (6.7%)
71-80	22	11	33 (2.7%)
81-90	6	4	10 (0.8%)
Total	680	520	1200

The mean total time taken from registration to the completion of phlebotomy was 9.8 minutes (Table 2). The maximum time was spent at step 2, i.e., the waiting time.

**Table 2:** Time in minutes for the three steps of phlebotomy.

	Step 1	Step 2	Step 3
Description	Registration/entry of patient details in the phlebotomy center register	Wait time in the queue to reach the phlebotomy station	Actual phlebotomy procedure time
Meantime in minutes	1.1 min	6.5 min	2.2 min
Median	1.150	6.417	2.083
2SD	0.7508	2.160	0.8060
Variance	61.38%	33.22%	36.40%

Different factors were identified as affecting the process, like apprehensive children and infants, passing instructions for proper collection of samples other than blood, anxious patients, difficulty in locating veins in patients due to thin veins in elderly and obese patients, differently abled and wheelchair-bound patients, etc. More time was naturally required for pediatric patients, as they were more apprehensive and likely to be uncooperative (Table 3). Many factors were identified as indirectly affecting the process; they have been listed in Table 4. Most of the factors for indirect effort were avoidable or of a resolvable nature, yet they seemed to be recurring and could not be eliminated.

**Table 3:** Direct factors affecting phlebotomy procedure time.

Factor	No. of cases	Percent (%)
Crying child as a patient	276	23%
Giving instructions about urine and sputum collection	168	14%
Allaying the anxiety of the patient	144	12%
Postprandial sample	144	12%
Difficulty in locating vein (in adults) with or without a second attempt for phlebotomy	84	7%
Patient syncopal/dizziness episode	36	3%
Physically challenged patient	24	2%
Random talk between phlebotomy staff or attending personal phones	24	2%
Wheelchair-bound patient	12	1%
Agitated patients breaking the queue	12	1%

**Table 4:** Indirect factors affecting phlebotomy procedure time.

Factor	Duration/average time lost (in min)	Average no. of times observed
Addressing billing problems	6	Three times per week
Unclear test request forms/overwriting	8	Two times per week
Queue management on and off	9	Two times per week
Washroom and water breaks	10	Two times per day
During planned and unplanned time out-inexperienced substitute phlebotomist	1.5 min for every patient	Two times per week
Replenishing phlebotomy trays	15 to 20 min	One time per week
Phlebotomy technicians sometimes have to carry samples to individual labs when the transporter is not available	10 min	One time per week

## Discussion

Phlebotomy is a manual technique that requires complete human attention, and it will probably never be fully automated, whatever the advances in artificial intelligence. It is the first step in any laboratory, and an incorrectly collected sample is bound to give erroneous results even with the most sophisticated analyzers [9]. It is much more than the venipuncture technique. Phlebotomists, in a very short time, have to build a rapport of trust and confidence with patients and draw blood samples in a skillful, safe, and professional way. The qualities of diligence, politeness, effective communication with patients, nurses, and other laboratory professionals, and the ability to work under pressure are expected of a phlebotomist [9].

Howanitz et al., in a large multicentric study, reported an average phlebotomy time of six minutes in approximately 50% of procedures. Less than five minutes were required in 25% of patients, and in 10% of cases, the blood draw required at least 21 minutes or even an hour, and almost one-third of the patients experienced more distress than expected. The average number of phlebotomy attempts per patient was 1.03, with 95 patients (0.4%) experiencing three to 11 attempts [10]. According to Jones et al. [11], the mean (SD) cycle time for phlebotomy was 259 seconds, i.e., 4.3 minutes. With expected minimum and maximum times (mean ± 2 SD), the time range is 196 to 404 seconds, i.e., 3.3 to 6.7 minutes. Our phlebotomy times were well within this limit.

Chung et al. [8] conducted a study in South Korea and observed that, on average, phlebotomy took one minute, 30 seconds per patient, and the waiting time for phlebotomy in their study was 7.3±4.3 minutes, accounting for 24.8% of pre-analytical phase. Our phlebotomy time was well within the limit. Melanson et al. [12] adopted Lean principles and observed that the elimination of non-value-added steps and modifications to operational processes resulted in increased capacity to handle workload during peak times without adding staff. Their average patient wait time was reduced from 21 to five minutes, and almost 90% of patients had a phlebotomy procedure performed within 10 minutes of reaching the phlebotomy center. They also observed increased patient satisfaction, which was assessed by a five-question survey.

Jeon et al. [13] from Korea observed that after introducing a system change, the average waiting time for phlebotomy reduced significantly to 2.34 minutes (the median was one minute), and the waiting time came to less than five minutes. This system change was in the form of the phlebotomist going to the patient actively rather than the patient waiting to be called by the phlebotomist. They also observed that the maximum number of patients seen by a phlebotomist during peak times also increased. Novis et al. [14] analyzed the results of three College of American Pathologists Q-Probes studies that surveyed the normative rates of laboratory technical staffing ratios at 67, 82, and 79 institutions in the years 2019, 2016, and 2014, respectively. They concluded that the technical staffing ratios vary widely among the various laboratory departments within each institution and also among different institutions.

Valenstein et al. [15] collected data from 151 clinical laboratories to study the staffing benchmarks for clinical laboratories. Data was collected for technical and management staffing and output from various sections like anatomic pathology, chemistry/hematology/immunology, microbiology, and transfusion medicine. They concluded that even though the testing methods in the clinical laboratory industry are standardized, there is still a wide variation in staffing levels among institutions. They suggest to look at this variation as an opportunity to improve staff productivity among the various facilities. A similar analogy is applicable to phlebotomy centers too.

The blood draw time has been variably reported in the literature. Leung et al. reported an average overall phlebotomy TAT of 23.4±4.1 minutes (SD) per phlebotomy request that included patient arrival to job completion [16]. Whereas, Howanitz et al. [10] in their study reported the median time required for phlebotomy as six minutes and observed that for 25% of patients, it required less than five minutes.

The estimated capacity of an outpatient phlebotomy center is determined by the formula: number of phlebotomists × time interval/service time per draw. For example, if the service time per draw was 10 minutes and three phlebotomists were working for 30 minutes, the estimated capacity of that center would be 3 × 30/10 = 9. That means that the center has the estimated capacity to draw nine patients in a 30-minute time interval [17].

According to the above formula, the estimated capacity of our center would be 4 × 300/9.8, i.e., 122.4 patients per day, i.e., approximately 30.6 patients in five hours per phlebotomist. Here, one hour of break time or time taken for hidden tasks has been considered for the six-hour shift, and draw time has been taken as 9.8 minutes. It was also observed that few patients arrived at the closing time of the center; their phlebotomy procedure spilled into the next half to one hour, followed by the winding up work, which took some more time. So sample collection center timing gets extended by almost an hour on a daily basis. Being aware of the above points will help in the adequate staffing of personnel. 

Novis et al. [18], in a large multicenter study, observed phlebotomy staffing in 40 selected inpatient sites and 70 selected outpatient sites. They reported the average outpatient wait time as four to 18 minutes, with a median average wait time of eight minutes.

As observed by Baisch [19], traditional methods would recommend seven technicians to complete a given work with their obvious or direct effort. However, the lab would need 12.6 technicians, of whom seven would do the actual direct effort and the remaining are required to handle the indirect and operational effort.

However, simply calculating the number of patients, the time taken to perform phlebotomy per patient, and working days per month won’t give us a correct idea of the optimum number of phlebotomists that are required. Overstaffing is a financial burden, whereas understaffing compromises the patient's test results and affects the quality of laboratory services. There has to be a balance between the number of people working and the work to be done.

While deciding on the adequacy of staff, one has to focus on three primary areas: direct effort, indirect effort, and operational needs. The direct effort is the actual labor effort related to hands-on time with the patient samples that transforms it from a specimen to a result [20]. 

Direct effort is the visible part of the labor in a laboratory and relates well with the number of patients, the number of samples, and the effort put into processing of samples. In the present study, it was observed that the actual phlebotomy procedure or the direct effort took only two to three minutes per patient, but various factors led to an increase in this time (Table 3). Also, rarely, some patients experience dizziness during phlebotomy, and they are asked to continue to be seated for a few more minutes till they feel better, which adds to the total phlebotomy time.

"Indirect effort" is most often invisible or hidden and refers to the labor effort that is not directly related to the patients, their samples, or the processing work but keeps happening in the background and has a bearing on the test results. Examples of indirect effort in a phlebotomy center are taking stock of supplies and related paperwork, attending to phone calls related to test queries, unplanned maintenance, time taken for any planned or unplanned inspections, billing issues, etc. In the present study, the "indirect efforts" that contributed to time loss were addressing billing problems, and in some instances, the phlebotomist had to leave his station and go to the billing section to resolve the issue. Other factors were unclear request forms and overwriting, making it unclear as to which exact samples were to be drawn. In some instances, the phlebotomists themselves had to do queue management when the security staff was unavailable or carry the samples to individual labs when the transport personnel were unavailable. Though most of these reasons are largely avoidable or easily addressable, they require good coordination between phlebotomy staff, security staff, and housekeeping staff. However, such occurrences are not uncommon and have to be kept in mind while considering staffing needs. To account for indirect effort, one has to consider all tasks that are done on a daily, monthly, and/or yearly basis but do not directly touch samples, test data, or patients but are nevertheless extremely important. Hence, while considering staffing, adequate staff has to be recruited to account for this time put in for indirect effort. 

The third is operational needs, which are defined as "non-procedural-based responsibilities that consume staffing resources." For example, compensatory off-duty for duty done on public holidays, i.e., planned time off or sudden unplanned emergency leave, wherein another substitute phlebotomist has to work, is likely to contribute to delays in various steps of sample collection. Also, technicians’ routine training schedules, training of new technicians where the existing technician has to teach, watch, and monitor the new technician till he/she gets used to the workflow, washroom breaks, and other short breaks.

It may appear unreasonable to hire additional staff when there is no apparent need for them. But "minimum staffing needs" should always consider any emergencies, any sudden surges in patients, which could be seasonal or otherwise, and also all the indirect and operational needs, which are hidden. Overstaffing has the advantage of being ready for any situation but increases the cost to the organization. Understaffing has the advantage of reduced short-term costs but leads to increased costs in the long term by way of delays and discomfort in patient care. Often, phlebotomists are preoccupied with sample collection only and underestimate the problems associated with improper sample collection, which leads to pre-analytical errors such as hemoconcentration, spurious hyperkalemia, and spurious hemolysis [21]. The following recommendations may be followed to streamline the process effectively.

Phlebotomy

Each phlebotomist in a six-hour shift should not exceed 30 to 35 phlebotomies. All items required in the phlebotomy center should be replenished on the previous day's afternoon itself. All hospital staff (doctors, residents, nursing officers, billing staff, technicians, etc.) should be aware of the tests available, their price lists and the prerequisites for sample collection, to minimize time loss while addressing these issues during active working hours. Proper queue management at all times has to be ensured by the hospital administration. Instruction boards about urine, stool, semen, and sample collection should be displayed prominently in the phlebotomy center to save technicians time.

Managerial

Work can be assigned based on high-volume days and low-volume days, i.e., Monday, Tuesday, and Saturday can be considered high-volume days, and Wednesday, Thursday, and Friday can be considered low-volume days. It is recommended that an extra hand from individual laboratories of pathology, microbiology, and biochemistry be added to the phlebotomy center on high-volume days. The "indirect effort" tasks such as stock taking, etc., should be reassigned to low-volume days. Coordination with clinicians regarding any research project work-related samples should be assigned to low-volume days. Coordination with the nursing officer in-charge for standby or backup of nursing personnel is essential. Taking input from the technical staff before making changes to their schedules is important, as they can provide first-hand information on the likely difficulties that may arise and also provide solutions for smoother functioning. Their view on staffing analysis should also be considered.

Each phlebotomy center is unique and may have its own requirements. What suits best to one center may not work for the other and hence, one has to understand the dynamics of each situation and strive for optimum balance.

## Conclusions

Adequate number of trained and effective phlebotomists is the first step in ensuring the success of any laboratory service, and while deciding on this "adequate number," not only the direct effort, but also the indirect effort, operational needs and emergencies have to be kept in mind. Though each phlebotomist in a six-hour shift can comfortably attend to 30 to 35 outpatients, the ideal phlebotomist to OPD patient ratio is 1:31 for a six-hour shift. When this number exceeds, additional staff has to be added. The study helps to assess the balance of the phlebotomist-to-patient ratio and provides a basis for the modification of the number of phlebotomists in order to ensure optimal patient service. This hospital-based study contributes to a better understanding of phlebotomy services in general and highlights the importance of practical methods of staffing in sample collection centers. 
